# Comprehensive Palliative Care in Patients with Lung Cancer Admitted to an Acute Palliative Care Unit

**DOI:** 10.3390/cancers18060886

**Published:** 2026-03-10

**Authors:** Sebastiano Mercadante, Gianfranco Mancuso, Yasmine Grassi, Alessio Lo Cascio, Alessandra Casuccio

**Affiliations:** 1 Main Regional Center for Pain Relief and Supportive/Palliative Care, La Maddalena Cancer Center, 90146 Palermo, Italy; grassi.yasmine@lamaddalenanet.it; 2 Lung Cancer Unit, La Maddalena Cancer Center, 90146 Palermo, Italy; mancuso.gianfranco@lamaddalenanet.it; 3 Direction of Health Professions, La Maddalena Cancer Center, 90146 Palermo, Italy; 4 Department of Health Promotion, Maternal and Infant Care, Internal Medicine and Medical Specialties, University of Palermo, 90146 Palermo, Italy; alessandra.casuccio@unipa.it

**Keywords:** lung cancer, acute palliative care unit, dyspnea, survival

## Abstract

**Background/Objectives**: The primary objective of this study was to assess the symptom profile and changes observed at discharge of lung cancer (LC) patients following comprehensive palliative care. The secondary objective was to evaluate potential differences between LCr patients and those with other cancer (OC) diagnoses. **Methods**: A consecutive sample of LC patients admitted to the acute palliative care unit (APCU) was prospectively assessed and compared with a random sample of patients with OC. All patients underwent comprehensive palliative care treatment. Demographic data, Karnofsky, referral sources, recent oncological treatments, and patient status at admission and discharge (on-treatment, off-treatment, or uncertain) were collected. At admission (T0) and at the time of discharge (TX), symptom burden was assessed using the Edmonton Symptom Assessment Scale (ESAS). Lastly, there was subsequent referral to next care settings (discharge home, home palliative care, hospice, other units). **Results**: A total of 159 patients with LC were compared with a similar sample of OC. In all patients a significant decrease in the number of “on therapy” patients were reported at discharge, and concomitantly the number of “off-therapy” patients increased (*p* < 0.0005) in comparison with the data recorded at admission. Dyspnea intensity was higher in group LC at T0 and TX (*p* < 0.0005), as well as pain intensity, which was significant at TX (*p* < 0.0005). A statistical difference in MDAS was also observed at TX (*p* = 0.034). LC patients had a lower overall survival (*p* = 0.034). **Conclusions**: Comprehensive palliative care in APCU provided relevant changes in symptom burden, with the potential to prevent inappropriate admissions to other hospital units and to reduce costs associated with non-specialist interventions.

## Simple Summary

People with advanced lung cancer often experience severe symptoms, especially breathlessness and pain, and may be admitted to hospital when problems become difficult to manage. Specialized acute palliative care units can provide intensive symptom control and help patients and clinicians make clearer decisions about whether cancer treatments should continue or be stopped. In this study, we examined lung cancer patients admitted to an acute palliative care unit and compared them with a similar group of patients with other cancers. We measured symptoms at admission and at discharge and recorded where patients went next (home, home palliative care, hospice, or another hospital unit) and whether anticancer treatment was continued. Symptoms improved substantially by discharge, although breathlessness remained more prominent in lung cancer. Many patients were discharged with a shift towards palliative-focused care. These findings support the role of acute palliative care units in improving symptom relief and guiding appropriate care pathways.

## 1. Introduction

Lung tumors are among the most prevalent and deadly forms of cancer worldwide [1]. The prognosis for lung cancer (LC) varies significantly depending on the stage at diagnosis. Unfortunately, many cases are diagnosed at an advanced stage, where the disease has already progressed and metastasized, leading to a poorer prognosis [2].

As the disease advances, symptoms tend to worsen, significantly impacting patients’ quality of life. The advanced phase of LC is characterized by significant disease progression, often involving metastasis beyond the lungs to other organs [3]. At this stage, treatment options become more limited and less effective, with curative approaches generally no longer feasible [1]. Patients frequently experience worsening symptoms, including persistent cough, chest pain, shortness of breath, fatigue, and weight loss. The progression of the disease can lead to complications that impair vital functions and substantially diminish quality of life [4].

In such cases, palliative care becomes essential to manage symptoms, improve comfort, and support both patients and their families through the challenges of the illness. Addressing the needs of individuals with advanced LC requires a multidisciplinary approach focused not only on extending survival but also on enhancing the quality of remaining life. Managing symptoms and providing supportive care are central components of treatment strategies during this stage, aiming to alleviate suffering and preserve the patient’s dignity [5,6,7]. Palliative care plays a crucial role in addressing the complex physical, psychological, and emotional needs of individuals with advanced LC, focusing on symptom control, psychological support, and improving overall comfort [8].

Palliative care has evolved rapidly, driven by an expanding body of clinical knowledge and the implementation of innovative models. These shifts have led to significant advancements in both practice and patient outcomes [9]. Such progress underscores the necessity of integrating palliative services beyond traditional home or hospice settings. In particular, Acute Palliative Care Units (APCUs) facilitate early intervention at any stage of the disease trajectory, rather than restricting palliative support to the final weeks of life [10].

These units serve as specialized hubs for managing patients with advanced diseases, addressing complex clinical complications, and reassessing treatment strategies, including continuation, modification, or discontinuation of oncological therapies. APCUs provide timely, multidisciplinary palliative care, facilitating rapid therapeutic adjustments based on continuous clinical assessments, imaging studies, and specialist consultations. Given the limitations of busy oncological units, often lacking beds or resources, APCUs offer a more appropriate setting for such interventions. Moreover, APCUs can prevent unnecessary admissions to other hospital units, reducing costs associated with non-specialist interventions [11].

It is essential to distinguish the Acute Palliative Care Unit (APCU) from other palliative care models. While hospices mainly provide comfort-focused care near the end of life and outpatient palliative services offer longitudinal support alongside oncology, an APCU is a high-intensity inpatient setting within (or closely integrated with) a comprehensive cancer center, designed to stabilize acute symptom crises (e.g., severe dyspnoea, refractory pain, delirium, uncontrolled nausea/vomiting) and manage complex transitions through rapid specialist review, frequent reassessment, and advanced pharmacological interventions, including parenteral titration when indicated [12]. This “acute symptom control” model targets short-term, high-complexity needs via structured monitoring and rapid optimization of symptom-control plans [13]. In comprehensive cancer care, specialists in inpatient palliative settings can act as a key “crossroad” in the oncological pathway, supporting timely reorientation of treatment intent, proportionality of interventions, avoidance of non-beneficial escalation, and coherent discharge planning across disease phases [14]. Integrated supportive and palliative care models emphasize early, needs-based access to specialist expertise, independent of proximity to death, with intensive inpatient care essential when symptom burden and clinical instability exceed what routine outpatient follow-up or ward-based advice can safely deliver [15].

In line with this paradigm, the American Society of Clinical Oncology (ASCO) clinical practice guidelines recommend early integration of specialist palliative care as a standard component of high-quality oncology care for patients with advanced cancer, including lung cancer, irrespective of life expectancy, in order to improve quality of life and symptom burden and to support appropriate goal-concordant decision-making along the disease course. Within this framework, APCUs can be understood as a service-level response that enables timely and guideline-concordant intensification of palliative interventions when acute complexity emerges during active treatment, at progression, or near the end of life [16,17].

In practical terms, APCUs address severe physical symptoms alongside psychological and existential distress, which can amplify suffering and complicate symptom control [13]. Compared with traditional inpatient palliative consultation services, where delivery can be influenced by variability in uptake, competing priorities of primary teams, and the absence of a protected environment for rapid iteration, dedicated APCU care provides concentrated expertise and continuous monitoring that can facilitate timely treatment adjustments and coordinated decision-making [18]. Observational evidence from comprehensive cancer centers indicates that APCU admission can support stabilization and appropriate downstream trajectories, with many patients being discharged to suitable settings, highlighting the unit’s operational role in crisis management and transition planning in advanced cancer [19]. At the hospital-system level, the opening of an APCU has been associated with improvements in administrative outcomes in general oncology wards, consistent with better alignment between patient needs and the care setting, and potentially more efficient patient flow [20]. Finally, economic evaluations of inpatient palliative care units report favorable cost signals, including cost savings over initial years of implementation, supporting the health-service rationale for APCU models when compared with less intensive or more fragmented approaches [21].

These units meet the comprehensive needs of cancer patients by addressing physical and psychological issues at any stage of disease, whether during active treatment or in advanced stages, helping to redirect care pathways, prevent overtreatment, and manage end-of-life care effectively. Multiple studies have reported that APCUs are associated with improved patient outcomes and cost savings compared to traditional palliative care consultation services.

## 2. Materials and Methods

This study is a preplanned secondary analysis of a larger investigation assessing outcomes in patients admitted to an Acute Palliative Care Unit (APCU). The protocol received approval from the local ethics committee (Comitato Etico Locale di Palermo 1), and informed consent was obtained from all participants. The research was conducted within a 12-bed APCU, a facility dedicated to teaching and research that has been operational for over 25 years within a comprehensive cancer center. Detailed characteristics of this unit have been previously described elsewhere [13,14]. Administratively and clinically, the unit is integrated with a 10-bed hospice located in an adjacent wing, as well as a comprehensive home palliative care program.

### 2.1. Patients

Over a 13-month period, we prospectively evaluated a consecutive cohort of oncology patients admitted to the APCU, specifically identifying those with a diagnosis of Lung Cancer (LC). All participants received integrated palliative care, characterized by ongoing symptom monitoring and therapeutic strategies tailored to individual patient needs. At the point of discharge, clinical status was reevaluated through oncological consultations and, where appropriate, diagnostic imaging. These assessments informed subsequent care pathways, such as the continuation of oncological therapy, treatment cessation, or transition to home-based or hospice palliative care.

### 2.2. Data Collection

Baseline characteristics, including demographic profile (age, sex), primary diagnosis, and performance status, were documented upon admission. We recorded referral origins, which spanned home settings, hospital departments, outpatient clinics, and other medical facilities. Furthermore, we tracked oncological interventions (chemotherapy, immunotherapy, or targeted therapy) administered within the 30 days prior to admission.

Patients were categorized based on their treatment status: ‘on-therapy’ for those currently receiving active anticancer treatment; ‘off-therapy’ for those who had ceased treatment due to disease progression, poor performance status, or toxicity; and ‘uncertain’ when a definitive therapeutic decision was pending. To quantify symptom burden, the Edmonton Symptom Assessment Scale (ESAS), a validated 0–10 metric [22] was utilized at admission (T0) and at discharge or the day preceding death (TX). Finally, the destination of discharge (e.g., home, hospice, or other units) was recorded. This study adheres to STROBE guidelines for observational research, and a randomized cohort of patients with other cancer (OC) diagnoses was used as a comparative group.

### 2.3. Statistics

Statistical processing was performed using IBM SPSS Software (version 24; IBM Corp., Armonk, NY, USA). Continuous and categorical data are reported with 95% confidence intervals (95% CI) and relevant effect sizes. To account for potential confounding variables, such as the Karnofsky Performance Status (KPS) and recent oncological history, a multivariable analysis was conducted. Survival probabilities were estimated via Kaplan–Meier curves. For all analyses, a two-sided *p*-value ≤ 0.05 was established as the threshold for statistical significance

## 3. Results

Of 520 patients admitted to an APCU during the study period, 159 (30.5%) had a diagnosis of lung cancer (LC). A similar sample (OC) was matched for age and gender and was selected for comparison. The general characteristics of the two groups, the change in setting at referral and discharge from the APCU, treatments received in the last 30 days, as well as treatments at admission and treatments proposed at discharge, are reported in Table 1.

In all patients, a significant decrease in the number of “on therapy” patients was reported at discharge, and concomitantly, the number of “off-therapy” patients increased (*p* < 0.0005) in comparison with data recorded at admission. In group LC, more patients were considered “uncertain” to be candidates for chemotherapy in comparison with group OC.

In both the LC and NL groups, symptom intensity at discharge improved significantly across all ESAS domains (*p* < 0.0005). However, dyspnea intensity was higher in group LC at T0 and TX (*p* < 0.0005), as well as pain intensity, which was significant at TX (*p* < 0.0005). Similar differences in the number of patients reporting an intensity of ≥4 were observed for dyspnea at T0 (37 v 18, *p* = 0.004) and TX (38 v 18, *p* = 0.038) and for pain at TX (38 v 18, *p* = 0.006).

A statistical difference in MDAS was also observed at TX (*p* = 0.034). Changes in ESAS, opioid doses, expressed as OME, and MDAS are reported in Table 2.

**Figure 1:**
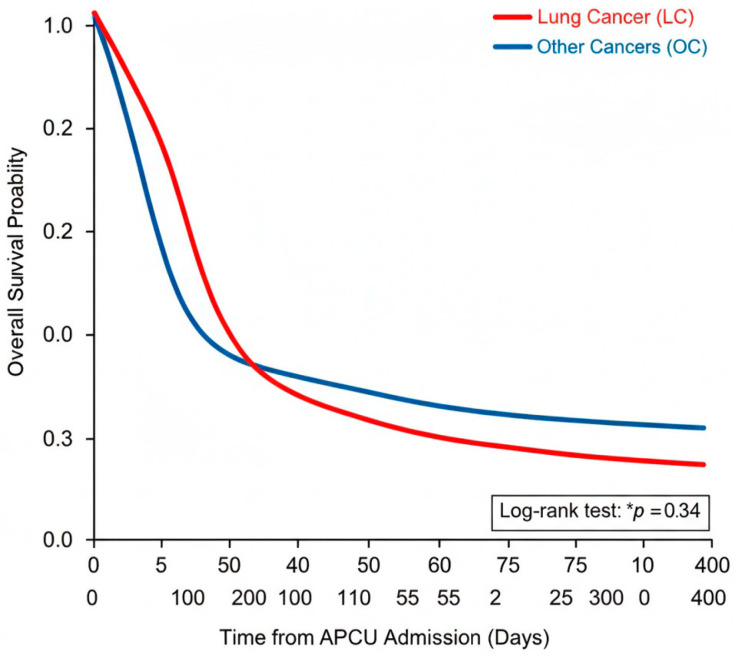
Kaplan–Meier Survival Curves.

Survival was available in 110 and 105 patients in groups LC and OC, respectively. Kaplan–Meier survival analysis showed that LC patients had a mean overall survival of 47 days (SD 116), which was significantly lower compared to the OC group (87.5 days, SD 158) (*p* = 0.034) (Figure 1).

## 4. Discussion

The present study provides insights into the characteristics of patients with LC admitted to an APCU at a comprehensive cancer center. Our findings suggest that while oncologists and acute care teams are often focused on disease-modifying treatments, they may lack the specific training or the necessary time to manage the complex transition to palliative care. Our data reflect a critical gap in standard oncology care: while acute care teams are dedicated to disease-modifying treatments, they often lack the specific training or dedicated time to navigate complex prognostic transitions. The APCU team does not simply act as a ‘harbinger of unpleasant news’; it provides a specialized clinical framework for Shared Decision Making. This process is a measurable intervention that ensures treatment proportionality, prevents futile aggressive care, and aligns medical strategies with the patient’s goals, which is a fundamental therapeutic outcome in modern oncology.

While some may argue that palliative interventions lack traditional measurable outcomes, our experience in the APCU demonstrates that the ‘outcome’ in this setting is the successful management of complexity. This includes not only the stabilization of acute symptoms but also the qualitative achievement of ‘Shared Decision Making’. As noted in the recent literature and in international guidelines (e.g., ASCO), the integration of these qualitative clinical experiences is essential for providing high-value care to lung cancer patients, who face a particularly high symptom burden and rapid prognostic transitions.

A large proportion of patients admitted “on therapy” were discharged “off-therapy,” and a considerable number of LC patients were discharged as “uncertain”. When combined with the observed increases in discharges to specialized palliative home care and hospice, these results indicate a transition toward palliative care. Accordingly, the APCU is a critical nexus for decisions regarding transitions to specialized palliative care in hospice or home settings and for the de-escalation or cessation of anticancer therapies. APCU admission permits reassessment, including imaging and bedside consultations with oncologists, enabling well-founded decisions on withdrawing anticancer treatments. This frequently leads to a substantial alteration in the clinical trajectory. Indeed, approximately two-thirds of patients who were on active therapy at admission remained candidates for ongoing treatment, highlighting the APCU’s role in improving clinicians’ ability to resume anticancer therapies when feasible.

A comprehensive palliative care treatment after admission to APCU resulted in a large improvement in symptom burden. However, in LC there was a higher intensity of dyspnea at admission, which was still evident at discharge compared with OC. This data is expected, given the common site of the disease, pre-existing comorbidity, and the symptom. Of interest, this symptom improved after comprehensive palliative treatment, as occurred with pain intensity. Moreover, more patients with LC were declared to be off-therapy at discharge after consultation between the palliative care and LC units, reflecting a more advanced stage of disease, comorbidity, and exhaustion of treatments. This is also confirmed by the finding of a lower overall survival in LC, possibly due to the presence of dyspnea as a leading symptom [23]. The median survival of 47 days in the LC group indicates that many lung cancer patients are still referred to palliative care close to the terminal phase. This finding highlights the need for ‘early integration’ to ensure patients benefit from palliative support long before the end-of-life stage. The primary reasons for APCU admission in LC patients were uncontrolled dyspnea, often related to pleural effusion or pneumonia, and severe pain, whereas acute vascular events like pulmonary embolism were less frequent.

Information about patients with LC admitted to an APCU is lacking, while some data exists on referrals to other palliative care services. In a study of inpatients and home care patients, the most frequent symptoms in both patient groups were dyspnea, fatigue, weakness, constipation, and anorexia. At the second evaluation, pain and weakness worsened in inpatients, while dyspnea was more intense in home-care patients [24]. In the US, a study showed a high proportion of patients experienced fatigue, loss of appetite, and pain, associated with poor quality of life [25].

Similarly, in a European study of patients with advanced lung cancer receiving anticancer treatments, fatigue, loss of appetite, dyspnea and pain were reported by most patients. They were found to be significant predictors of lung cancer-specific quality of life [24].

A comparative analysis of patients referred to a palliative care outpatient clinic revealed a distinct clinical profile: these individuals exhibited elevated scores for pain, fatigue, anorexia, and general symptom distress compared to non-referred patients. Furthermore, this cohort was characterized by a more compromised performance status and a significantly reduced survival rate [26].

Supporting these findings, a cross-sectional study identified fatigue (85.5%), sleep disturbances including daytime somnolence (73.5%), and emotional distress (73.0%) as the most prevalent symptomatic clusters among patients with LC. When evaluating the burden of these symptoms, the most profound impact was observed in areas of social role dissatisfaction, functional impairment, and persistent fatigue. Notably, disability status emerged as the strongest predictor of symptom prevalence. Additionally, advanced disease (Stage IV), active oncological therapy, and a history of smoking were significantly correlated with an increased frequency of these symptomatic themes [6].

In a randomized controlled study with combined early palliative care, newly diagnosed patients with lung cancer lived longer, had better quality of life, were psychologically stable, were in less pain, and were more nutritionally satisfied [7].

These studies, however, were performed in different settings, stages of disease, or were cross-sectional, and did not focus on symptom control and pathway direction, which can be performed in an APCU [19,27,28]. In this longitudinal study, comprehensive palliative care given in an APCU provided effective symptom control in patients with LC. Patients with advanced LC have a poor prognosis, but both chemotherapy and early palliative care have been shown to improve survival and quality of life.

Among patients with advanced LC, early palliative care may optimize patient selection for chemotherapy receipt by focusing on quality of life in accordance with patients’ performance, preferences and goals of care [5]. Among 149 decedent patients, 50% of them met a palliative care team, and the median time from the first encounter to death was 2.3 months. In the last month of life, at least one criterion of aggressive care was present for 70% [29]. In a retrospective study, 43% of patients seen for the first time at an LC clinic were referred to an outpatient palliative care clinic within a median of 30 days, and 80% of them received simultaneous anticancer therapy and palliative care. Referral to the clinic was associated with worse performance status, more advanced disease stage, pain, dyspnea, and cough [30].

The main limitation of this study stems from its single-center experience. Our single-center design may limit the generalizability of the findings reported within a high-volume APCU, which admits more than 500 patients annually and is affiliated with a 10-bed hospice operated by the same clinical team. This hospice primarily serves patients no longer eligible for anticancer therapy as well as those with other incurable non-cancer conditions. The geographical proximity and prior team familiarity likely facilitated transitions to hospice beds, potentially contributing to the comparatively low death rate observed in this APCU. Conversely, patients with imminent death are typically not transferred to hospice and remain in the unit in a single-occupancy room to enhance privacy for the family of the dying patient. The characteristics of patients who die in the unit have been detailed in a subanalysis [22].

A subset of patients admitted to hospice care experiences clinical stabilization, allowing for a feasible discharge to their homes. Consequently, the transfer to a hospice facility should not be considered a primary driver of APCU mortality rates [31]. To the best of our knowledge, this specific integrated care model has not been extensively documented in the existing literature.

A potential limitation of the current study is the lack of a direct comparator, such as palliative care consultation services or standalone home-based programs. However, conducting such comparisons remains inherently complex due to significant variations in patient demographics and institutional resources. To further validate these findings and determine the necessity of the APCU model within tertiary care centers, future multicenter research and larger-scale investigations across diverse clinical settings are warranted. Furthermore, while the APCU model suggests a potential for resource optimization, we did not perform a direct cost-effectiveness analysis. Future studies should include patient-reported quality-of-life outcomes beyond the ESAS scores.

In summary, an APCU staffed by a specialized multidisciplinary team provides comprehensive palliative care for patients with Lung Cancer (LC), enabling swift clinical decision-making and dynamic therapeutic adjustments. This is achieved through continuous observation, expert specialist evaluations, and rigorous monitoring of high-complexity cases. Compared to the high-pressure environment of a standard oncology ward, the APCU model facilitates a more structured admission process and optimizes the overall care trajectory for this patient population.

Our findings suggest that the APCU functions as a critical clinical nexus, potentially diverting inappropriate admissions from other hospital departments and reducing the costs associated with non-specialist interventions. Beyond direct patient admission, the APCU team serves as a vital resource for the entire hospital, providing expert consultations and influencing care standards across various units. To validate these single-center preliminary results, further research in similar facilities with comparable resources is essential. Despite these advancements, integrated palliative care remains underutilized for patients with LC. It is therefore imperative that cancer centers systematically monitor key performance indicators, including the timing of palliative care involvement and the aggressiveness of end-of-life interventions.

## 5. Conclusions

In this prospective cohort subanalysis, comprehensive palliative care delivered in an acute palliative care unit (APCU) was associated with clinically meaningful improvement in overall symptom burden from admission to discharge in patients with lung cancer, mirroring benefits observed in a matched cohort with other malignancies. Despite significant symptom relief across Edmonton Symptom Assessment Scale domains, dyspnoea remained more prominent in lung cancer at both time points, underscoring the need for targeted respiratory symptom strategies within APCUs. Admission to the APCU also facilitated timely reassessment of anticancer treatment appropriateness, with a marked shift from “on-therapy” to “off-therapy” status and a substantial proportion of lung cancer patients discharged as “uncertain”, reflecting complex decision-making near the end of life. Lung cancer patients had shorter overall survival than comparators, consistent with higher baseline and residual dyspnoea. These findings support APCUs as pivotal hubs for symptom control and care pathway optimization in advanced lung cancer.

## Figures and Tables

**Table 1:** Characteristics of two patient groups, referral/discharge setting ratio, treatment in the previous 30 days, and proposed anticancer treatment.

Baseline Characteristics			
Variable	LC (159 pts)	OC (159 pts)	*p*
Age (yrs, mean SD)	67.5 (10.5)	67.4 (10.5)	-
Gender (M/F)	89/70	89/70	-
Karnofsky (mean SD)	43.3 (10.3)	43.9 (10.1)	0.587
**Primary tumor**	**LC (159 pts)**	**OC (159 pts)**	
Lung	159	0	
Breast	0	30	
Genitourinary	0	26	
Gastrointestinal	0	73	
Hematologic	0	4	
Head-neck	0	1	
Unknown	0	16	
Other	0	9	
**Referral/Discharge**			
**Setting**	**LC (Referral/Discharge)**	**OC (Referral/Discharge)**	***p***
Home	108/61	102/59	
Home palliative care	22/31	24/39	
Hospice	0/37	0/33	
Surgery unit	14/0	12/0	
Oncology unit	11/0	11/1	
Other hospitals	4/5	7/7	
Hematology unit	0/0	3/0	
Long staying	0/3	0/4	
Radiotherapy	0/1	0/0	
Died in APCU	21	16	
**Overall *p***			**0.539/0.809**
**Treatments in the previous 30 days**			
**Treatment**	**LC**	**OC**	***p***
Chemotherapy	58	56	
Target therapy	4	3	
Immunotherapy	4	2	
Hormonal therapy	0	5	
Radiotherapy	5	1	
**Overall *p***			**0.123**
**Proposed anticancer treatments**			
**Treatment status**	**LC (Admission/Discharge)**	**OC (Admission/Discharge)**	***p***
On	72/46	65/49	
Off	43/83	39/79	
Uncertain	4 */10	19 */16	
Naïve	26	33	
**Overall *p***			**0.001/0.515**

* intergroup difference.

**Table 2:** Comparison of intensity of ESAS items, opioid dosage, and MDAS (means, SD) between two patient groups.

	LUNG	OTHERS		LUNG	OTHERS	
	Mean (SD) T0	Mean (SD) T0	*p* Inter Group *	Mean (SD) TX	Mean (SD) TX	*p* Inter Group *
Pain	4.0 (2.8)	3.7 (2.9)	0.383	2.3 (1.8)	1.2 (1.8)	<0.0005
Dyspnea	2.1 (2.7)	0.9 (1.9)	<0.0005	0.9 (1.7)	0.3 (1.0)	<0.0005
Anxiety	2.9 (3.0)	2.8 (3.1)	0.724	1.4 (2.2)	1.4 (2.2)	0.910
Depression	2.2 (2.9)	2.6 (3.1)	0.228	1.1 (2.2)	1.2 (2.2)	0.485
Poor sleep	3.9 (3.2)	3.9 (3.2)	0.812	2.0 (2.7)	1.9 (2.4)	0.859
Drowsiness	2.7 (2.5)	2.7 (2.7)	0.761	1.6 (2.0)	1.7 (2.1)	0.899
Nausea	0.7 (2.1)	1.0 (2.2)	0.178	0.3 (1.1)	0.2 (1.0)	0.409
Poor appetite	4.2 (3.8)	3.7 (3.3)	0358	1.9 (2.7)	1.8 (2.7)	0.609
Weakness	5.9 (2.7)	5.8 (2.9)	0.915	3.5 (2.6)	3.3 (2.8)	0.509
Poor well-being	5.3 (2.9)	4.8 (2.9)	0.123	3.0 (2.4)	2.4 (2.6)	0.058
Global ESAS	33.5 (14.4)	31.9 (15.7)	0.344	17.9 (13.3)	15.6 (12.6)	0.102
OPIOIDS (OME)	101 (106)	77 (83)	0.099	87 (101)	73 (161)	0.447
MDAS	3.4 (3.3)	3.4 (2.9)	0.580	1.9 (3.1)	2.6 (3.9)	0.034

* Mann–Whitney U statistic test.

## Data Availability

The original contributions presented in this study are included in the article. Further inquiries can be directed to the corresponding authors.

## Abbreviations

The following abbreviations are used in this manuscript:

APCU	Acute Palliative Care Unit
ESAS	Edmonton Symptom Assessment Scale
LC	Lung Cancer
OC	Other Cancers
MDAS	Memorial Delirium Assessment Scale
OME	Oral Morphine Equivalent
SD	Standard Deviation
T0	Admission (baseline)
TX	Discharge (or day before death)
